# Trends in Ecological Research during the Last Three Decades – A Systematic Review

**DOI:** 10.1371/journal.pone.0059813

**Published:** 2013-04-24

**Authors:** Yohay Carmel, Rafi Kent, Avi Bar-Massada, Lior Blank, Jonathan Liberzon, Oded Nezer, Gill Sapir, Roy Federman

**Affiliations:** 1 Faculty of Civil and Environmental Engineering, Technion – Israel Institute of Technology, Haifa, Israel; 2 Department of Plant Sciences, University of Cambridge, Cambridge, United Kingdom; 3 Department of Biology and Environment, University of Haifa at Oranim, Kiryat Tivon, Israel; 4 Institute of Evolution, University of Haifa, Haifa, Israel; Northwestern University, United States of America

## Abstract

It is thought that the science of ecology has experienced conceptual shifts in recent decades, chiefly from viewing nature as static and balanced to a conception of constantly changing, unpredictable, complex ecosystems. Here, we ask if these changes are reflected in actual ecological research over the last 30 years. We surveyed 750 articles from the entire pool of ecological literature and 750 articles from eight leading journals. Each article was characterized according to its type, ecological domain, and applicability, and major topics. We found that, in contrast to its common image, ecology is still mostly a study of single species (70% of the studies); while ecosystem and community studies together comprise only a quarter of ecological research. Ecological science is somewhat conservative in its topics of research (about a third of all topics changed significantly through time), as well as in its basic methodologies and approaches. However, the growing proportion of problem-solving studies (from 9% in the 1980s to 20% in the 2000 s) may represent a major transition in ecological science in the long run.

## Introduction

Ecologists often describe ecological science as dynamic. ‘Ecology is a science in transition’ [1]. This transition is characterized by several significant shifts in emphasis and perspective [2]. During most of the 20^th^ century, the majority of ecologists conceptualized ecological systems as balanced and stable, typically at equilibrium, or as returning to such equilibrium deterministically following rare disturbances [3]. In recent decades, there has been a shift towards an understanding of ecological systems as nonlinear, constantly changing, and unpredictable in time and space [4], [5]. The concept of equilibrium was replaced by other concepts, for example, the concept of non-equilibrium change, in which the system is often described as rotating between alternative states [6].

Ecologists are split on the question of whether the changes in ecological science represent a Kuhnian ‘paradigm shift’ [5], [7], [8], [9], or, alternatively, a gradual accumulation of modifications, better characterized as ‘evolution’ rather than ‘revolution’ [2], [10]. In contrast, other ecologists maintained that progress in ecology is lacking [11] or limited [12].

Here, we ask if the topics and methodologies of ecological research as reflected in the literature of the last 30 years provide evidence to support notions of dramatic shifts, or of gradual change. We characterize various aspects of ecological research, using an extensive survey of ecological literature. In particular, we ask three questions regarding general aspects of ecology, and look for possible changes in these aspects over the last 30 years:

- Domains of ecological research: What proportion of research is devoted to the various domains in ecology (population, species, community, and ecosystem)? What are the major topics of ecological study? Has there been a change in the frequency of investigation of any of these topics and, if so, which ones?
- Types of research: Is ecology an experimental science, or a science of observation and measurement? How often are models used in ecological research? To what degree do ecologists use meta-analysis of data from previous studies (vs. collecting new data in each research)?
- Basic science or problem-solving oriented discipline: Is ecology becoming a problem-solving science? In other words, how often does ecology relate to actual, specific environmental problems, in an attempt to provide solutions (or at least new insights on how to make progress towards solutions)?

### Preliminary expectations

#### A. Domains of ecological research

The concepts of *ecosystem* and *community* have become increasingly dominant in ecological thinking. In a survey conducted among members of the British Ecological Society, *ecosystem* was identified as the single most important concept in ecology [13]. More recently, the Ecological Visions Committee of the Ecological Society of America issued a report that listed eight critical environmental issues for prioritizing ecological research [14]. Only two of those topics related to populations and species, while five topics were clearly within the domains of ecosystems and communities**.** We expected an increase in research conducted at the ecosystem level, and at the community level, accompanied by a proportional decrease in studies of single species. We also expected specific topics to become more frequent subjects of ecological study (such as biodiversity, climate change, biogeochemistry, and scale).

#### B. Types of research

Observations and experiments are known to be the two dominant tools of ecological research**.** In this research, we expected to identify an increase in the frequency of models, for two reasons: (1) the ecosystem has increasingly been described as ‘complex’, and models are often the only tools available for the study of complex systems, and (2) due to the substantial increase in the availability of modelling tools during the last three decades. We also expected an increase in the proportion of meta-analysis studies, for two major reasons: (1) a growing awareness of the incapacity of single studies of specific systems, conducted under narrow ranges of conditions, to provide insights on broader ecological issues [15], and (2) the increased access to information and data in the age of the Internet.

#### C. Is ecology a problem-solving science?

In the past, ecologists have been reluctant to engage in applied research [16]. Applied science was considered inferior to basic, ‘pure’ science [17]. Some applied ecological issues, such as conservation, are emotionally charged [2], and perceived by some ecologists as ‘advocacy’ [18]. More recently, ecologists have become increasingly concerned about the implications of their work to society's problems [15], [17], while environmental agencies have expressed an increased demand for ecological solutions to environmental problems [19]. For these reasons, we expected to find an increase in the proportion of applied studies over the last three decades.

In order to attempt to answer these questions, a quantitative survey of ecological research is required. Surprisingly, few attempts have been made to systematically quantify trends in ecological research. Typically, these studies have used an automated count of words in titles and abstracts to assess trends in ecology [20], [21], [22], [23]. Shorrocks [24] used an alternative method to survey trends in ‘the Journal of animal ecology’ –he actually sampled 13 volumes of the journal between 1932 and 1992. Here, we followed that method: we inspected a large sample of the ecological literature, classifying it according to its content. This process is time-consuming, but the resulting analysis is probably more reliable than an automated word count.

## Methods

### Two surveys

One major consideration was our choice of target population within the ecological literature. Two plausible alternatives existed: we could either sample the entire pool of ecological research, or sample only leading journals. There are pros and cons to each choice. Including the entire range of ecological literature may introduce research of varying quality into the analysis. On the other hand, niche journals (the vanguard of novel research) may serve as early indicators of transitions and trends. We therefore decided to conduct two parallel surveys, using identical methods. In survey 1, we included all 136 journals that concern ecology, while in survey 2 we sampled eight ‘core journals’ that were published throughout the entire study period. A brief description of the data collection approach can be found in the Prisma 2009 flow diagram and checklist.

### Journal selection

For survey 1, we selected all relevant journals that appeared during at least parts of the study period 1981–2010. This pool consisted of 136 journals. From the entire collection of articles published in these journals during this period, we limited the selection to research articles in English, and received a total of 110,965 articles (see Appendix S1 for a full list of journals sampled for this survey).

For survey 2, we selected eight prominent journals, using the following criteria: (a) high-impact factor (among the top 30 ecological journals, using ISI Web of Science Impact Factor), (b) generality (cover the entire scope of ecological research), and (c) consistency (were published throughout the study period). Not a single journal satisfied all three criteria. We therefore selected eight journals belonging to three major ecological societies that issue their own journals; thus, each group, as such, satisfies all three criteria. The eight journals were those issued by the Ecological Society of America ( *Ecological Applications*, *Ecological Monographs*, and *Ecology*); the British Ecological Society (Journal of Ecology, Journal of Animal Ecology, and Journal of Applied Ecology), and the Nordic Ecological Society (*Oikos* and *Ecography*). *Ecological Applications*, first published in 1991, was an offshoot of Ecology, and *Ecography*, first published in 1991, was an offshoot of *Oikos*; we assumed that the range of topics covered by each of the pairs was similar to that of the parent journal prior to the split. The pool of all research articles published in these journals in the period 1981–2010 consisted of 22,788 articles).

For each of the two surveys, we used a random selection scheme to select 25 articles from each year, totalling 750 articles in each survey. The classification (domain, topics, research type, applied or basic science) was performed by the authors of the current study, based on the articles. In many cases, reading the abstract provided sufficient information for classifying the article. In order to ensure a high degree of consistency between the classifiers, we carried out a pilot exercise, in which the degree of agreement between the classifiers was assessed prior to the research study. A set of 29 articles was classified independently by all classifiers. Classifications were then discussed until consensus was reached for each classification. For each topic and for each classifier, the level of agreement between initial classification and final ‘consensus’ was recorded.

### Article characterization

#### A. Ecological domains

We predefined 20 topics that describe major research fields in ecology, and grouped these 20 topics into five broad ecological domains: (1) Single Species (demography, physiology, distribution, behaviour, evolution, genetics); (2) Species Interactions (grazing, predation, mutualism, parasitism, competition); (3) Community (biodiversity, community structure); (4) Ecosystem (food web, climate change, vegetation dynamics, biomass and productivity, biogeochemistry); and (5) Other topics (scale, statistics). We limited topic-based characterization to three topics per article.

#### B. Type of research

We classified the type of ecological research according to four general categories: experiment, observation, model, and data analysis. An article was classified as ‘experiment’ if an actual experiment was conducted in the laboratory, or if a field study included some sort of treatment or manipulation of the natural environment. Where research included both observation and field experiment, the article was labelled ‘experiment’. ‘Observation’ was a study where the major activity was any sort of measurement of ecological phenomena. An article was labelled ‘model’ if its sole activity or the major endeavor was to construct a model. In cases where a model was only a minor part of the research, the article was labelled ‘experiment’ or ‘observation’. Articles that did not present any new data, but used data collected in previous studies, often conducting meta-analysis, were labelled ‘data analysis’. Articles that did not include any of the above types of research, but discussed ecological issues qualitatively were omitted from the survey (and a replacement was added).

#### C. Problem-solving

Our goal here was to determine the degree to which ecology is oriented towards problem-solving. We assigned the category of ‘application’ to all articles that either searched for solutions to problems associated with anthropogenic activities, or proposed tools for practical problems (such as practices for conservation, global change mitigation etc.).

### Statistical analyses

The number of articles assigned to each ecological topic, label, and variable was recorded for each survey. The differences between surveys in terms of the frequency of each term were analyzed using Chi square test. To evaluate change in the frequency of these variables over time, we used logistic regression [25], with publication year as a continuous variable and survey type as a fixed variable. In order to account for multiple comparisons, we applied the Bonferroni correction. Fifty comparisons (25 comparisons in each survey) yielded a threshold of *p<0.001*. The Bonferroni correction becomes very conservative when the number of comparisons becomes large, as it controls the probability of false positives only, at the cost of increasing the probability of false negatives [26]. We therefore report the results using Bonferroni correction, as well as for less conservative thresholds.

## Results

### Classification consistency

Classifiers' results were in good agreement with the consensus of the test articles, with an overall average agreement rate of 90%. The average accuracy of parameter classification was high in all cases, ranging from 86% for ‘topics’ and ‘problem solving’, to 93% and 94% for ‘research type’ and ‘domain’, respectively. In what follows, wherever we report two figures, the first figure refers to the ‘all journals’ survey, and the second figure refers to the ‘core journals’ survey.

### Domains of ecological research

(1) Single Species was the most frequent domain of study in this survey of ecological research, with 71% (66%) of all the studies involving topics within this domain (Table 1). In both surveys, the four most common topics related to this domain: demography, physiology, behaviour and distribution. Taken together, these topics appeared in 64% (63%) of all articles. Only 4% (3%) of all articles studied evolution. (2) In the Species Interactions domain, predation and competition were the frequent terms, recorded in 5–9% of the articles, while mutualism and parasitism were recorded in 2–4% of the articles. (3) Community-related topics (biodiversity and community structure) appeared in 17% of the studies in both surveys, and (4) Ecosystem-related topics appeared in nearly a quarter of the articles. Among ecosystem topics, biogeochemistry accounted for 11% (8%) of ecological research and 2% of the research concerned climate change studies (Table 1).

**Table 1:** Frequency of domains and topics in ecological research 1980–2010.

		Frequency of Topics (%)		
Domain	Topic/Type	All Journals	Core Journals	
**Single Species**	Demography	123 (16.4)	219 (29.2)	***
	Physiology	230 (30.7)	154 (20.5)	***
	Distribution	113 (15.1)	98 (13.1)	
	Behavior	139 (18.5)	96 (12.8)	**
	Evolution	31 (4.0)	23 (3.0)	
	Genetics	76 (10.1)	15 (2.0)	***
Single Species		532 (70.9)	498 (66.4)	
**Species Interactions**	Grazing	17 (2.3)	69 (9.2)	***
	Predation	53 (7.1)	66 (8.8)	
	Mutualism	12 (1.6)	16 (2.1)	
	Parasitism	20 (2.7)	35 (4.7)	*
	Competition	37 (4.9)	53 (7.1)	
Species Interactions		132 (17.6)	224 (29.9)	***
**Community**	Biodiversity	72 (9.6)	58 (7.7)	
	Community structure	68 (9.1)	82 (10.9)	
Community		124 (16.5)	127 (16.9)	
**Ecosystem**	Food web	25 (3.3)	12 (1.6)	*
	Climate change	15 (2.0)	13 (1.7)	
	Vegetation dynamics	30 (4.0)	89 (11.9)	***
	Biomass and productivity	60 (8.0)	28 (3.7)	***
	Biogeochemistry	81 (10.8)	63 (8.4)	
Ecosystem		121 (16.1)	135 (18.0)	
Others	Scale	7 (0.9)	20 (2.7)	*
	Statistics	11 (1.5)	17 (2.3)	

Differences between the two surveys are: *** significant at the Bonferroni-adjusted level, p<0.001, ** p<0.01, * p<0.05.

**Figure 1:**
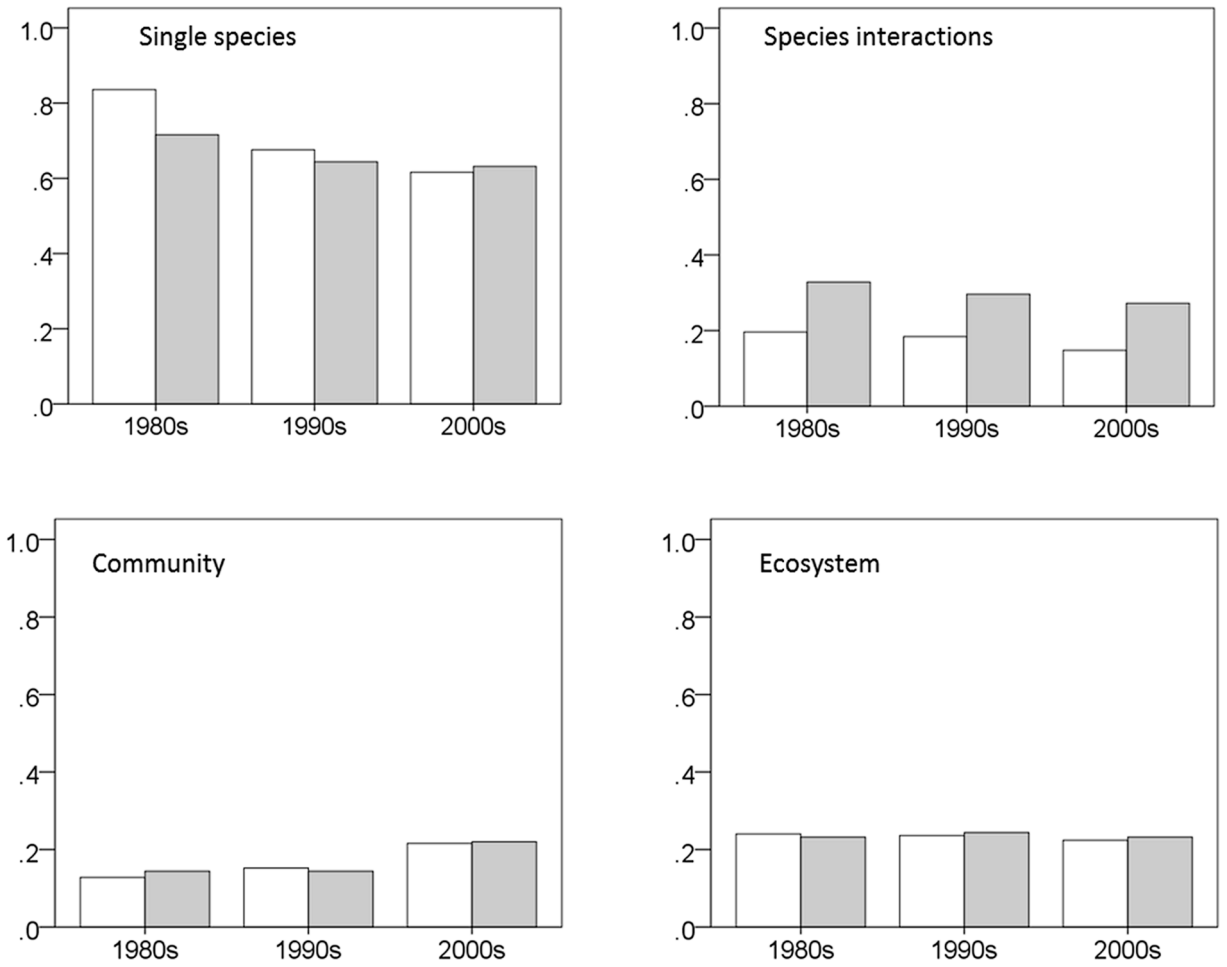
Proportions of ecological domains in the last three decades. White bars denote ‘all journals’ and gray bars denote ‘core journals’. Temporal trend was significant for community studies only (a logistic model, see Table 2).

The frequency of community studies increased significantly (nearly significantly in the ‘core journals’) during the studied period, while the other three domains remained quite constant over time (Table 2, Figure 1).

**Table 2:** Annual percentage change in the frequency of domains and topics in ecological research 1980–2010.

		Percent of annual average change (95% CI)		
Domain	Topic	All Journals	Core Journals	
**Single Species**	Demography	NS	−2.0 (−0.2– −3.8)*	
	Physiology	−3.1 (−5.9– −1.3)***	NS	
	Distribution	NS	NS	
	Behavior	−5.0 (−7.1–−2.8)***	NS	
	Evolution	NS	5.9 (0.2–11.9)*	
	Genetics	10.4 (6.9–14.1)***	NS	
Single species		NS	NS	
**Species Interactions**	Grazing	NS	−3.2 (−6.0–−0.3)*	
	Predation	NS	NS	
	Mutualism	NS	NS	
	Parasitism	NS	5.2 (0.9–9.7)*	
	Competition	NS	NS	
Species interactions		NS	NS	
**Community**	Biodiversity	6.0 (2.8– 9.2)***	4.7 (1.4–8.2)**	
	Community structure	NS	NS	
Community		3.6 (1.3–6.0)***	2.9 (0.6–5.3)**	
**Ecosystem**	Food web	NS	NS	
	Climate change	7.7 (0.8–15.0)*	13.9 (4.5–24.2)***	
	Vegetation dynamics	NS	−2.7 (−5.2–−0.1)*	
	Biomass and productivity	NS	NS	
	Biogeochemistry	NS	NS	
Ecosystem		NS	NS	
Others	Scale	NS	9.0 (2.7–15.7)**	
	Statistics	NS	NS	

*** Change is significant at the Bonferroni-adjusted level, p<0.001, ** p<0.01, * p<0.05.

**Figure 2:**
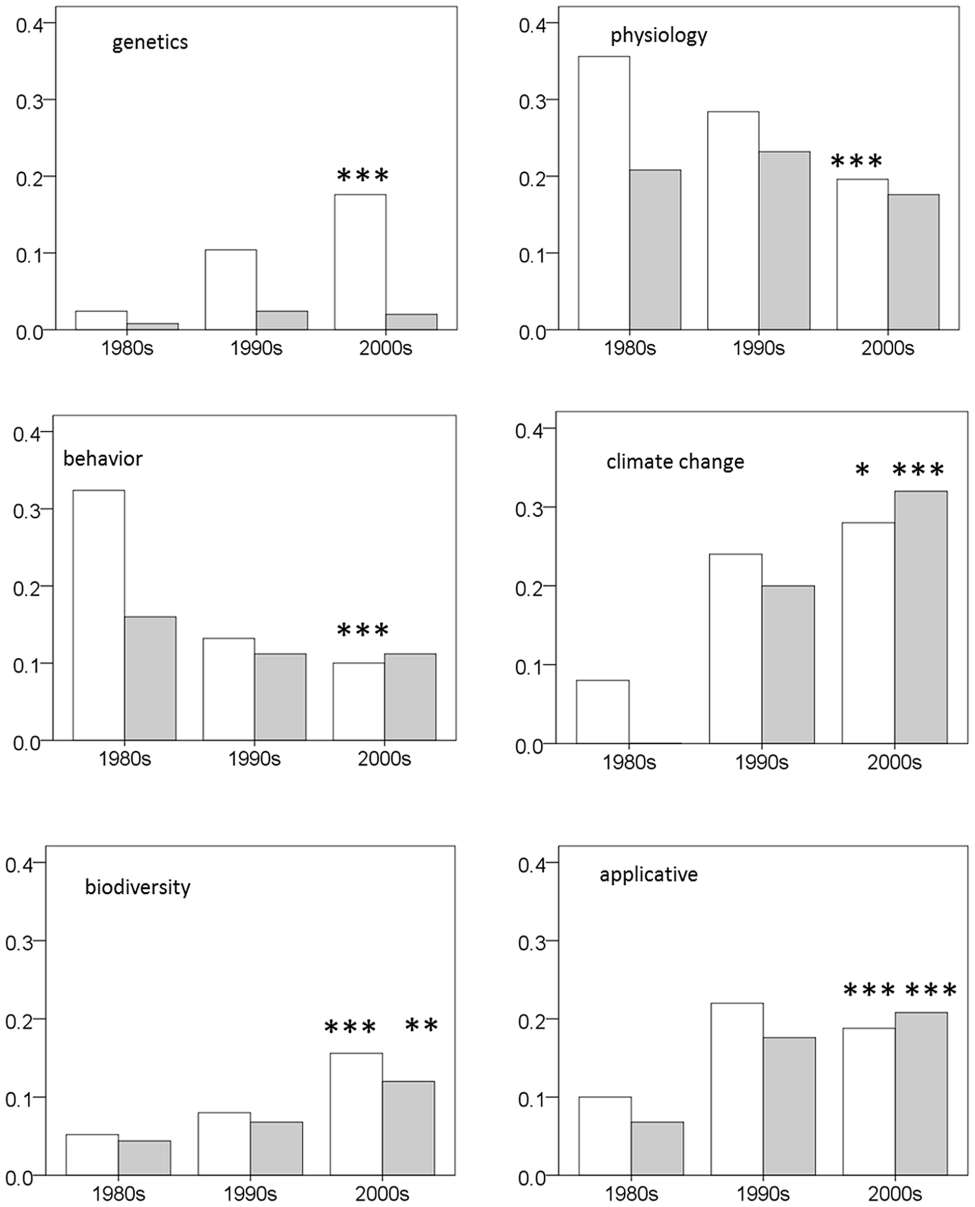
Change in the frequency over time for the topics for which temporal change was significant at the Bonferroni-adjusted level (p<0.001) in at least one of the datasets. White bars denote ‘all journals’ and gray bars denote ‘core journals’. *** Temporal trend was significant (a logistic model, see Table 2), p<0.001. ** p<0.01. * p<0.05.

There were significant changes in the frequency of several topics over time. The frequency of two topics climate change and biodiversity, increased significantly with time in both surveys (Table 2, Figure 2). The frequency of three additional topics changed significantly in the ‘all journals’ survey only: *physiology* and *behaviour* decreased, while *genetics* increased. An increase in the frequency of *scale* was the single significant change that appeared in the ‘core journals’ only. Additionally, five topics revealed a nearly significant frequency change through time in that survey (p<0.05): *demography*, *grazing*, and *vegetation dynamics* decreased, while *evolution* and *parasitism* increased in frequency with time (Table 2).

### Differences between the two surveys

The results of both surveys were quite similar for 14 of the 20 topics, while significant differences between the two surveys were found for six topics: *physiology*, *behaviour* and *genetics* were much more frequent in the ‘all journals’ survey, while *demography*, *grazing*, and *vegetation dynamics* were much more frequent in the ‘core journals’ survey (Table 1). Most domains appeared at similar frequencies in the two surveys, except Species Interactions, which was nearly twice as frequent in the ‘core journals’ survey compared to its frequency in the ‘all journals’ survey (Table 1).

### Type of research

Observations constitutes the major type of ecological research (59%, 45%), followed by experiments (28%, 36%), while models (12%, 12%) and data-analysis (9%, 6%) were less frequent (Table 3).

**Table 3:** Frequency of research types and problem-solving studies, in ecological research 1980–2010.

	Frequency (%)		
Research Type	All Journals	Core Journals	P
Observation	446 (59.4)	334 (44.5)	***
Experiments	206 (27.5)	264 (35.2)	**
Models	89 (11.9)	87 (11.6)	
Data Analysis	66 (8.8)	46 (6.1)	*
Problem solving	127 (17.0)	113 (15.1)	

Differences between the two surveys are: *** significant at the Bonferroni-adjusted level, p<0.001, ** p<0.01, * p<0.05.

The proportion of data-analysis studies increased significantly with time in the ‘all journals’ survey. The use of models as a primary research tool slightly decreased in ‘all journals’ and slightly increased (nearly significant) in the ‘core journals’ survey (Table 4).

**Table 4:** Annual percentage change in the frequency of research types and problem-solving studies, in ecological research 1980–2010.

	Percent of annual average change (95% CI)	
Research Type	All journals	Core Journals
Observation	NS	NS
Experiments	NS	NS
Models	NS	3.5 (0.8–6.2)**
Data Analysis	4.9 (1.7–8.1)**	NS
Problem Solving	4.7 (2.3–7.1)***	5.5 (3.0–8.1)***

*** Change is significant at the Bonferroni-adjusted level, p<0.001, ** p<0.01, * p<0.05.

Observation studies were significantly more frequent in the ‘all journals’ survey, while experiments were somewhat more frequent in the ‘core journals’ survey.

### Is ecology a problem-solving science?

Overall, 17% (15%) of the articles were labelled ‘problem solving’ (Table 3). In both surveys, their proportion increased significantly over time, from 9% (7%) in the 1980s to 21% (21%) in the 2000 s (Table 4, Figure 2).

## Discussion

Few systematic surveys of ecological literature have been conducted to date, and most have been restricted to a single theme or a narrow branch of ecological science [20], [21], [22], [23]. For example, [22] evaluated relations between the size of the organism and its relative representation in ecological research. Swihart [12] quantified the rates of appearance of new ecological terms and disappearance of old terms. Shorrocks [24] was perhaps the only investigator to quantify various trends in ecological science, using articles published in The Journal of Animal Ecology between 1932 and 1992. To the best of our knowledge, the present study is the first attempt to systematically survey the entire breadth of ecological literature, in order to quantify various characteristics of the science of ecology, as well as their temporal trends. The results suggest that ecology may be substantially less dynamic than is generally acknowledged.

### Domains of ecological research

Ecology is mostly a study of single species. Most of the ecological research focused on the demography, physiology and distribution of single species. The proportion of single-species studies has slightly decreased in the past three decades, but still consists of more than 60% of the studies. In comparison, community and ecosystem studies represented a minor fraction of ecological research. This surprising finding seems at odds with the strong emphasis on the community and the ecosystem as major concepts in ecology [27], [28]. Also surprising was the scarcity of a few topics which are thought to be central in ecology. Two notable examples are evolution, and food-web, each of which appeared as a research topic in 2–4% of the articles. Most of the increase in community studies occurred in the 2000s, probably reflecting the renewed interest in this field, after the neutral theory challenged the prevalence of the niche concept.

The analysis of changes in the frequency of research topics over time provided inconclusive results. Only two topics, climate change and biodiversity, showed a significant change in both surveys. The increase in both topics probably relates to the fact that both were non-issues at the beginning of the period under study. Four other topics changed significantly, and seven other topics changed nearly significantly, in only one of the surveys. Overall, there does not seem to be a drastic transformation in the relative importance of domains and topics in the field of ecology, but the apparent change in topics and research types signifies that ecological science is not entirely stagnant.

The frequency of more than half of the topics and domains was very similar in both surveys, but nearly a third of the topics differed significantly between the surveys. Interestingly, the topics that were significantly more frequent in the ‘all journals’ survey related to the basic and static aspects of a species (genetics and physiology), and the ecosystem (biomass and productivity). In contrast, the topics that were significantly more frequent in the ‘core journals’ related to dynamic processes (demography, vegetation dynamics, and grazing).

### Type of research

Observation and experiment were by far the predominant tools of ecological study, together accounting for 80% of the research; these proportions did not change over time. Interestingly, modelling (∼12% of all studies), is no more common today than it was thirty years ago, despite a drastic increase in the availability of modelling tools during this period. Data-analysis became a more common research tool. Many of the studies in this category were, in fact, meta-analyses (analyses of data from several sources). The major increase in data-analysis studies was in the mid-90s, suggesting that the increased availability of information in the age of the Internet had an important role in this trend.

Comparing the two surveys in terms of type of research revealed a fundamental difference: the ratio of experiments to observations in the ‘all journals’ survey was 1:2, while in the ‘core journals’ survey it was 7:9. The prevalent consensus that ecology has changed during the 20^th^ century, from an observational to an experimental science, may be somewhat overstated; nevertheless, such a change appeared more prominently in the ‘core journals’ survey.

### Is ecology a problem-solving science?

Ecological research is mostly a basic science, with only a small proportion of ‘problem solving’ studies. Yet, in both surveys we found a significant and consistent increase in the number of ‘problem solving’ articles published during the survey period. If this trend continues in future decades, it may prove to be a major shift in the orientation of ecology.

### Is ecology a dynamic science?

Prominent ecologists have claimed that ecology has undergone transitions [29], and even paradigm shifts [5] in recent decades, and is now a mature and competent science [30]. Our survey reveals that these claims perhaps overstate the case. The science of ecology appears to be changing slowly, in the sense that major research subjects and principal methodologies have not changed dramatically for at least 30 years. In particular, the popular image of ecology as a science in transition [7], dealing chiefly with ecosystems and communities [1] seems at odds with the major proportion of single species studies reported here.

A contrasting view, put forward by O'Connor [11], claimed that ecology lags after other life sciences, and makes very little progress. O'Conner's study ignited a debate, wherein various arguments were employed to disprove this claim [31] ^,^ [23], or put it in a balanced perspective [12]. This debate is still ongoing, and is probably driven by emotions no less than by objective evaluations. The current study does not substantiate O'Connor's claim, and it was not meant to evaluate progress. However, it is safe to assume that a major advance in ecology would be accompanied by a major change in the frequency of domains, topics, and types of research; yet, as shown here, these have changed only moderately in the course of three decades.

A major aspect of progress in science is the rate at which basic questions in ecology are being answered [12], which we have not evaluated, and is very difficult to evaluate quantitatively. Also, we could not detect conceptual shifts, such as network thinking, that do not connect to particular terms or topics. Swihart et al. [12] provide an interesting attempt to quantify progress based on ‘birth rate’ and ‘death rate’ of ecological terms, and claim to show viable progress in ecology. In contrast, the list of 100 fundamental questions in ecology [32] reports profound knowledge gaps regarding the central mechanisms driving ecosystems, communities, and even population dynamics.

Our approach could not, and was not meant to detect changes in particular methods and technologies applied within each research domain or topic. The availability of advanced molecular and genetic tools and the increase in computing power have allowed analyses to become more complex and sophisticated. However, the use of these new technologies and processing power does not imply enhanced knowledge or understanding. Also, such surveys may not detect conceptual shifts, such as network thinking, which do not connect to particular terms or topics.

Perhaps the single and most important change in the study of ecology is the growing proportion of ecological research directed towards problem solving. This trend by itself, if continued, may represent a major transition in ecology in the long run.

Our results may be disturbing to some researchers, insofar as they portray an ecological discipline which is considerably less dynamic than ecologists would like to believe. The value of this research is precisely in reviving the debate and presenting an opportunity for self-assessment to those who strive to advance the discipline, all of which can serve to stimulate the investigation of new and groundbreaking tools, paradigms and perspectives. Only through meta-scale monitoring of the scope of research can we understand, and hope to influence, the trajectory of ecological research in the years to come.

## Supporting Information

Appendix S1 **A full list of journals sampled for survey 1.**
(DOCX)

Flow Diagram S1 (DOC)

Checklist S1 (DOC)
