# Supplementary material for: Trends in Ecological Research during the Last Three Decades – A Systematic Review
Source: PLoS One. 2013 Apr 24;8(4):e59813. doi: 10.1371/journal.pone.0059813 (PMC3634786; doi:10.1371/journal.pone.0059813)
Supplement: Appendix S1 — A full list of journals sampled for survey 1. (DOCX) [file pone.0059813.s001.docx]

**Appendix S1**. List of the 136 journals used in survey 1.

| Acta Ecologica Sinica | Advances in Ecological Sciences | Advances in Ecological Sciences |
| --- | --- | --- |
| African Journal of Ecology | Agriculture Ecosystems and Environment | American Midland naturalist |
| American Naturalist | Animal Conservation | Annual Review of Ecology and Systematics |
| Applied Ecology and Environmental Research | Applied Soil Ecology | Applied Vegetation Science |
| Aquatic Ecology | Aquatic Microbial Ecology | Austral Ecology |
| Australian Journal of Ecology | Australian Wildlife Research | Basic and Applied Ecology |
| Behavioral Ecology | Behavioral Ecology and Sociobiology | Biochemical Systematics and Ecology |
| Biodiversity and Conservation | Biological Conservation | Biotropica |
| BMC Ecology | Canadian Field Naturalist | Chemistry and Ecology |
| Chinese Journal of Applied Ecology | Chinese Journal of Ecology | Community Ecology |
| Conservation Biology | Conservation Ecology | Conservation Genetics |
| Diversity and Distributions | Ecography | Ecological Applications |
| Ecological Complexity | Ecological Economics | Ecological Engineering |
| Ecological Entomology | Ecological Indicators | Ecological Informatics |
| Ecological Management and Restoration | Ecological Modelling | Ecological Monographs |
| Ecological Research | Ecological Restoration | Ecologist |
| Ecology | Ecology and Society | Ecology Environment and Conservation |
| Ecology Letters | Ecology of Freshwater Fish | Ecoscience |
| Ecosystems | Ecotoxicology | Environment Ecology |
| Environmental Conservation | Ethology Ecology and Evolution | European Journal of Wildlife Research |
| Evolutionary Ecology | Evolutionary Ecology Research | FEMS Microbiology Ecology |
| Fisheries Management and Ecology | Forest Ecology and Management | Frontiers in Ecology and the Environment |
| Functional Ecology | Global Change Biology | Global Ecology and Biogeography |
| Holarctic Ecology | Human and Ecological Risk Assessment | International Journal of Ecology and Environmental Sciences |
| International Journal of Sustainable Development and World Ecology | Japanese Journal of Ecology | Journal of Animal Ecology |
| Journal of Applied Ecology | Journal of Arid Environments | Journal of Biogeography |
| Journal of Chemical Ecology | Journal of Ecology | Journal of Ecology and Rural Environment |
| Journal of Ecology China | Journal of Evolutionary Biology | Journal of Experimental Marine Biology And Ecology |
| Journal of Freshwater Ecology | Journal of Industrial Ecology | Journal of Range Management |
| Journal of Tropical Ecology | Journal of Vector Ecology | Journal of Vegetation Science |
| Journal of Wildlife Management | Landscape and Urban Planning | Landscape Ecology |
| Marine Biology | Marine Ecology | Marine Ecology Progress Series |
| Microbial Ecology | Microbial Ecology in Health and Disease | Molecular Ecology |
| Molecular Ecology Notes | Molecular Ecology Resources | Netherlands Journal of Aquatic Ecology |
| New Zealand Journal of Ecology | Oecologia | Oikos |
| Oryx | Paleobiology | Perspectives in Plant Ecology Evolution and Systematics |
| Physiology Ecology Japan | Plankton Biology and Ecology | Plant Ecology |
| Polar Biology | Polish Ecological Studies | Polish Journal of Ecology |
| Population Ecology | Rangeland Ecology and Management | Rangeland Journal |
| Researches on Population Ecology | Restoration Ecology | Russian Journal of Ecology |
| South African Journal of Wildlife Research | South African Journal of Wildlife Research | Southwestern Naturalist |
| Soviet Journal of Ecology | Terrestrial Ecology | Theoretical Ecology |
| Trends in Ecology and Evolution | Trends in Ecology and Evolution | Tropical Ecology |
| Urban Ecology | Wetlands | Wetlands Ecology and Management |
| Wildlife Biology | Wildlife Monographs | Wildlife Research |
| Wildlife Society Bulletin |  |  |
