# Supplementary material for: Trends in Ecological Research during the Last Three Decades – A Systematic Review
Source: PLoS One. 2013 Apr 24;8(4):e59813. doi: 10.1371/journal.pone.0059813 (PMC3634786; doi:10.1371/journal.pone.0059813)
Supplement: Flow Diagram S1 — (DOC) [file pone.0059813.s002.doc]

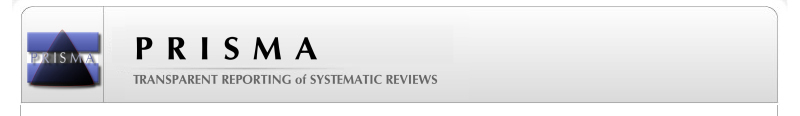
**PRISMA 2009 Flow Diagram**

**Screening**

**Included**

**Eligibility**

**Identification**

Records identified through database searching
(n =750 )

Additional records identified through other sources
(n = n/a )

Records after duplicates removed
(n = 750 )

Records screened
(n =750 )

Records excluded
(n = 0 )

Full-text articles assessed for eligibility
(n = 750 )

Full-text articles excluded, with reasons
(n = n/a )

Studies included in qualitative synthesis
(n = 750 )

Studies included in quantitative synthesis (meta-analysis)
(n = 750 )
